# Unraveling trends in schistosomiasis: deep learning insights into national control programs in China

**DOI:** 10.4178/epih.e2024039

**Published:** 2024-03-13

**Authors:** Qing Su, Cici Xi Chen Bauer, Robert Bergquist, Zhiguo Cao, Fenghua Gao, Zhijie Zhang, Yi Hu

**Affiliations:** 1 Department of Epidemiology and Biostatistics, School of Public Health, Fudan University, Shanghai, China; 2 Key Laboratory of Public Health Safety, Ministry of Education, Shanghai, China; 3 Xuhui District Center for Disease Control and Prevention, Shanghai, China; 4 Department of Biostatistics and Data Science, University of Texas Health Science Center at Houston, Houston, TX, USA; 5 Ingerod, Brastad, Sweden; 6 Anhui Institute of Parasitic Diseases, Wuhu, China; 7 Laboratory for Spatial Analysis and Modeling, School of Public Health, Fudan University, Shanghai, China

**Keywords:** Schistosomiasis, Deep learning, Spatio-temporal analysis, China

## Abstract

**OBJECTIVES:**

To achieve the ambitious goal of eliminating schistosome infections, the Chinese government has implemented diverse control strategies. This study explored the progress of the 2 most recent national schistosomiasis control programs in an endemic area along the Yangtze River in China.

**METHODS:**

We obtained village-level parasitological data from cross-sectional surveys combined with environmental data in Anhui Province, China from 1997 to 2015. A convolutional neural network (CNN) based on a hierarchical integro-difference equation (IDE) framework (i.e., CNN-IDE) was used to model spatio-temporal variations in schistosomiasis. Two traditional models were also constructed for comparison with 2 evaluation indicators: the mean-squared prediction error (MSPE) and continuous ranked probability score (CRPS).

**RESULTS:**

The CNN-IDE model was the optimal model, with the lowest overall average MSPE of 0.04 and the CRPS of 0.19. From 1997 to 2011, the prevalence exhibited a notable trend: it increased steadily until peaking at 1.6 per 1,000 in 2005, then gradually declined, stabilizing at a lower rate of approximately 0.6 per 1,000 in 2006, and approaching zero by 2011. During this period, noticeable geographic disparities in schistosomiasis prevalence were observed; high-risk areas were initially dispersed, followed by contraction. Predictions for the period 2012 to 2015 demonstrated a consistent and uniform decrease.

**CONCLUSIONS:**

The proposed CNN-IDE model captured the intricate and evolving dynamics of schistosomiasis prevalence, offering a promising alternative for future risk modeling of the disease. The comprehensive strategy is expected to help diminish schistosomiasis infection, emphasizing the necessity to continue implementing this strategy.

## GRAPHICAL ABSTRACT

[Fig f6-epih-46-e2024039]

## Key Message

Our research found that CNN-IDE model effectively captured the complex dynamic process of schistosomiasis prevalence. The comprehensive strategy is expected to help diminish schistosomiasis infection.

## INTRODUCTION

Schistosomiasis is a chronic parasitic disease associated with poverty, caused by blood flukes belonging to the genus Schistosoma [1,2]. Considered by the World Health Organization as a neglected tropical disease, schistosomiasis is mainly prevalent in low-resource tropical and subtropical areas, with an estimated 140 million cases worldwide in 2019 [3]. Three species of *Schistosoma* are commonly seen in Asia, with *Schistosoma japonicum* the most prevalent, followed by *S. mekongi* and *S. malayensis* [4]. *S. japonicum* has a history of more than 2,200 years in China [5]. Over the past 60 years, China has made significant strides in reducing the prevalence of schistosomiasis; currently, fewer than 29,000 people nationwide are estimated to be living with the disease, and only 5 new cases were reported in 2020 [6]. However, the risk of *S. japonicum* infection still exists in some areas of China, and the goal of eliminating the infection by 2030 remains a challenge [7].

Over the past 6 decades, China has made great strides toward reducing the prevalence of schistosomiasis. The World Bank Loan Project (WBLP) for schistosomiasis control and prevention from 1992 to 2001 made substantial progress; however, it focused on the treatment (e.g., praziquantel chemotherapy) [8] and not the transmission sources (e.g., intermediate snail host), and the epidemic rebounded following the end of the project. The integrated control strategy implemented in 2005, which focused on eliminating the intermediate snail host, again reduced the number of *S. japonicum* infections [9,10], and the prevalence of schistosomiasis in the country has now stabilized at a historical low.

In order to better understand the transmission patterns and the temporal trend of schistosomiasis, previous studies employed numerous spatio-temporal models to estimate the infection risk and the contributing factors. Most studies have used Kriging [11,12], regression techniques [13,14], or linear dynamic models (e.g., integro-difference equations [IDE]) [15]. However, the dynamic transmission patterns of schistosomiasis are not fully captured by descriptive or linear models due to the complexity of the process and the multitude of contributing factors, which include environmental changes, human behavior, and evolving interventions. Therefore, in this study, we have adopted a deep learning (DL) approach with the aim of capturing the intricate and dynamic patterns of the disease. Our analysis provides detailed information on the annual prevalence of schistosomiasis across a grid of 1× 1 km. This information is valuable for both researchers and local policymakers, enabling them to comprehend the evolution of schistosomiasis distribution patterns under the 2 national schistosomiasis control programs (NSCPs) and to identify areas in urgent need of disease intervention.

## MATERIALS AND METHODS

### Study area

**Figure 1.**
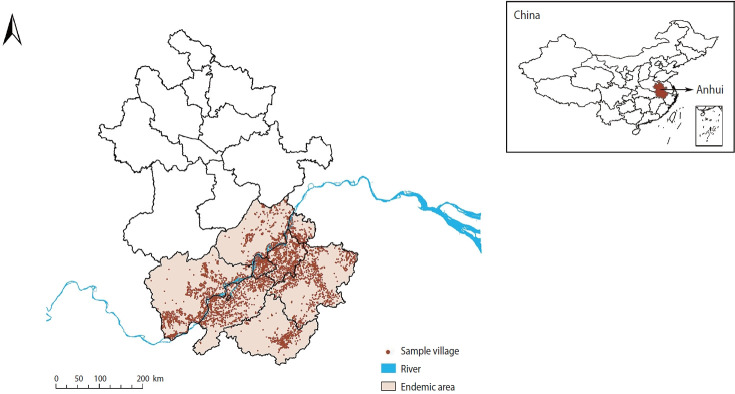
Endemic areas of schistosomiasis in Anhui Province, China. Anhui Province is located in the lower reaches of the Yangtze River in eastern China.

The study was conducted at the village level in Anhui Province, located in eastern China, where the Yangtze River traverses the province (Figure 1). Anhui Province covers a geographic area of approximately 140,100 km² and had a population of nearly 65 million in 2019 (http://tjj.ah.gov.cn/). The province experiences a humid subtropical climate, with average annual temperatures ranging from 15°C to 16°C. Summers are typically hot, with temperatures frequently exceeding 30°C, while winters are cooler, with temperatures often falling below 10°C. The region receives a significant amount of rainfall, averaging between 1,000 mm and 1,500 mm annually, with the heaviest precipitation occurring in late spring and early summer. Humidity levels are generally high, often surpassing 80% during the rainy season. These climatic conditions—high temperatures, ample rainfall, and elevated humidity—create an ideal environment for the proliferation of *Oncomelania hupensis*, the freshwater snail that serves as the intermediate host for *S. japonicum*. Therefore, snail populations increase during the warmer and wetter months, which in turn affects the transmission dynamics of schistosomiasis in Anhui Province.

### Parasitological data

Prevalence data for schistosomiasis from 1997 to 2015 were obtained from cross-sectional surveys conducted by the Anhui Institute of Parasitic Diseases. In China, schistosomiasis is classified as a Class B notifiable infectious disease, ensuring that case detection at the village level is comprehensive, with a coverage rate of 100%. Upon identifying a schistosomiasis case, healthcare providers are required to complete an infectious disease report card within 24 hours of diagnosis for network reporting. Data collection occurred annually at the village level, targeting the population aged 5-65. A 2-tiered diagnostic approach was employed: all participants were initially screened using the indirect hemagglutination assay [16], and positive readings were confirmed by Kato-Katz stool examinations [17] of all seropositive individuals. Individuals testing positive by both methods were diagnosed with *S. japonicum* infection. The study’s annual selection of sample villages was systematically conducted in accordance with the “Control and Elimination of Schistosomiasis (GB15976-1995)” guidelines established by the National Health Commission of the People’s Republic of China in 1995. Each year, villages categorized as level 1, with a human prevalence exceeding 5% in the previous year, were included in the study. In contrast, villages categorized as level 2, with a human prevalence below 5% in the previous year, were included every 2 years. Furthermore, villages categorized as level 3, indicating a human prevalence of less than 1% in the year before, were selected every 3 years. To ensure the accuracy of our disease prevalence calculations and maintain consistency with the methodologies of our previous studies [12,18], sample villages with fewer than 100 participants were omitted to mitigate the impact of statistical outliers. Throughout the duration of the study, the number of sample villages varied between 1,028 and 1,683. Detailed enumerations are provided in the Supplementary Material 1.

### Environmental data

Considering the environmental factors that affect snail habitats and the growth and reproduction of *O. hupensis*, the intermedia host snail of *S. japonicum*, we included the following covariates: precipitation, hours of daylight, distance to water bodies, daytime land surface temperature (LST_day_) and the normalized difference vegetation index (NDVI). We collected raster variable data for the corresponding years (1997-2015) for the areas included in the study, and the data for the distance to water bodies were from 2015. Average daily precipitation and hours of daylight were obtained from the China Meteorological Data Sharing Service System (http://data.cma.cn/site/article/id/51.html), from 610 weather stations across China. We performed Kriging interpolation to produce continuous overlays each year during the study period for all of China and then extracted the interpolated meteorological measures for Anhui Province using ArcGIS 10.5 (Esri, Redlands, CA, USA). Water-body data were downloaded from the World Wildlife Fund’s Conservation Science Data Sets (http://worldwildlife.org), and the Euclidean distances between the geographic centroids of each sampled villages and water bodies were calculated. The NDVI and LST_day_ data were obtained from NASA’s Level 1 and Atmosphere Archive and Distribution System (http://ladsweb.nascom.nasa.gov), which included 8-day 1-km^2^ images for LST_day_ and the monthly 1-km^2^ for NDVI. All the above data were processed using annual data and the raster data with a resolution of 1 km. The detailed sources of all the environmental data, as well as the resolution of time and space, are presented in the Supplementary Material 2.

### Statistical analysis

We treated the prevalence of schistosomiasis as a continuous variable and converted it to a Gaussian distribution, and the prevalence was transformed by the Box-Cox method [19] to satisfy the assumptions of a Gaussian model. We used univariate analysis for initial variable screening, and retained any variables with a p-value < 0.1 [20]. We then assessed the correlations among the remaining variables, where correlation coefficients > 0.6 indicated strong collinearity [21]. The 5 covariates we selected proved not to be colinear.

### Convolutional neural network

To evaluate the potential non-linear spatio-temporal trends of schistosomiasis and the influence of environmental factors, we considered a convolutional neural network (CNN) based on an IDE framework (i.e., CNN-IDE) [22] to model the non-linear trend. We employed a 2-level hierarchical structure [23] in the IDE model, with a data level and a process level. The former modeled the data generating mechanism, conditioned on the underlying spatial-temporal process and parameters, while the latter considered the unobserved process given by the parameters. Details on this CNN-IDE model can be found in the Supplementary Material 3.

### Model comparison and validation

In addition to the CNN-IDE, we also implemented 2 spatiotemporal models (IDE and ST Kriging [7]) to estimate the risk of *S. japonicum* infection. The IDE model was the same as the IDE framework in CNN-IDE. Similarly, we constructed a hierarchical structure including a data level and a process level. Detailed descriptions of the 2 models can be found in the Supplementary Material 3.

Cross-validation is used to evaluate model predictions by splitting the data into training and validation samples, then training the model with the training sample, and evaluating the model with the validation sample. In K-fold cross-validation, the available data are randomly divided into K-folds. Each fold is excluded, the model is trained on the remaining K−1 folds, and then the model is evaluated on the initially excluded fold. Specifically, for k= 1..., K folds, the model is fit after removing the Kth fold, and the prediction result ${\hat{Z_{v}}}^{\left(-k\right)}$ is obtained for *i*=1…*m_k_*, where *m_k_* is the number of data in the Kth fold. We conducted 10-fold cross-validation [23] in to evaluate the performance of the 3 models and to identify the optimal model. We used 2 evaluation indicators, the mean-squared prediction error (MSPE) and continuous ranked probability score (CRPS), which are defined as follows:


(1) $$MSPE=\frac{1}{Tm}\sum_{j=1}^{T}\sum_{i}^{m}{\left\{Z_{v}\left(s_{i};t_{j}\right)-\hat{Z_{v}}\left(s_{i};t_{j}\right)\right\}}^{2}$$

{*Z_v_(s_i_;t_j_)*} represent observations of a randomly selected 10% of samples, and $\hat{Z_{v}}\left(s_{i};t_{j}\right)$ are predictions from modeling the rest of the observations.


(2) $$CRPS=\int {\left(1\left\{Z_{v}\leq \hat{Z_{v}}\right\}-F\left(\hat{Z_{v}}\right)\right)}^{2}\;d\hat{Z_{v}}$$

Where $1\left\{Z_{v}\leq \hat{Z_{v}}\right\}$ indicates that if Z_v_ less than *x*, the value is `1. otherwise 0, and *F*() is the cumulative distribution function of hte observed 10% of samples, Smaller vallues of MSPE and CRPS indicate better model performance. Our analyses were all done in R version 3.6.3 (R Foundation for Statistical Computing, Vienna, Austria). A high-resolution spatial prediction (1 km^2^) of schistosomiasis prevalence was malled using the optimal model.

### Ethics statement

The collection of parasitological data was part of a continuing public health investigation determined by the National Health and Family Planning Commission. Hence, this study was exempt from institutional review board assessment.

## RESULTS

**Figure 2.**
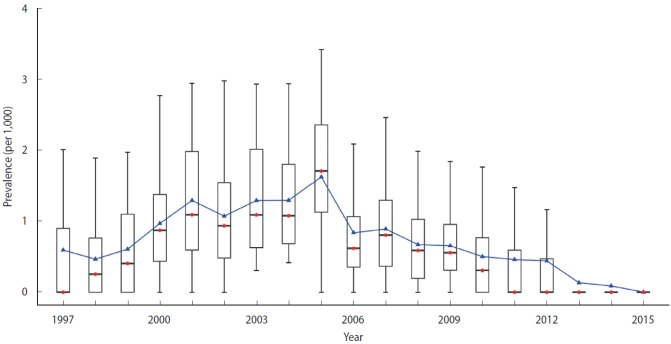
Box plot for the observed prevalence of schistosomiasis in sample villages in Anhui, China, from 1997 to 2015. The blue line represents the average annual prevalence, and the red points are the median annual prevalence. The boxes denote minimum, median, maximum, and interquartile ranges.

As shown in Figure 2, the median annual prevalence of schistosomiasis began to increase in 1997 and reached a peak in 2005 (1.6 per 1,000). There was a sharp decline to 0.6 per 1,000 in 2006, and the prevalence continued to decrease rapidly, approaching zero after 2010. The mean annual prevalence exhibited a similar pattern. The interquartile range widened from 0.0-0.8 (per 1,000) in 1997 to 1.2-2.4 (per 1,000) in 2005, then diminished to 0.0% by 2013.

**Figure 3.**
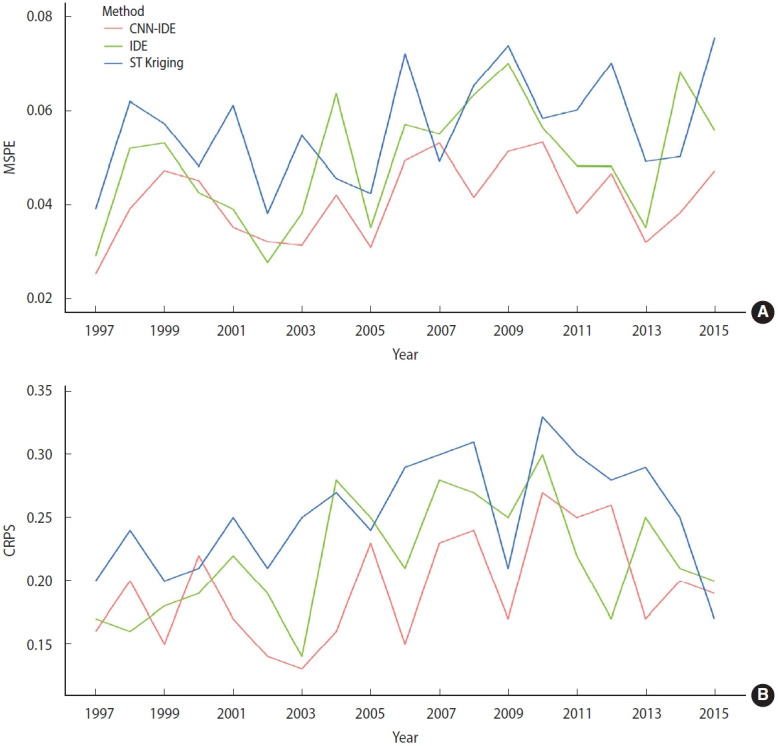
(A) Average mean squared prediction error (MSPE) and (B) continuous ranked probability score (CRPS) of the CNN-IDE predictions (red), the IDE predictions (green) and the ST Kriging predictions (blue) as a function of time. CNN, convolutional neural network; IDE, integro-difference equation.

Figure 3 presents the results of model comparison. As shown in the figure, both the MSPE and CRPS for the CNN-IDE model were lower than those from the other 2 models for most of years. The overall average MSPE values of the CNN-IDE model, the IDE model, and ST Kriging model were 0.04, 0.05, and 0.06, respectively, and the overall CRPS values were 0.19, 0.22, and 0.25, respectively.

Table 1 shows the final environmental covariates and parameters in the CNN-IDE model. The average daily precipitation (p= 0.02) and NDVI (p< 0.01) showed statistically significant positive associations with schistosomiasis prevalence, while LST_day_ (p< 0.01), with longer hours of daylight (p< 0.01), and distance to a water body (p< 0.01) exhibited statistically significant negative associations. The estimate for the diffusion parameter *θ*_*p*,1_ was 2.31E+02, and those for the shift parameters *θ*_*p*,2_, and *θ*_*p*,3_ were 6.50E-03 and 2.45E-02, respectively.

**Figure 4.**
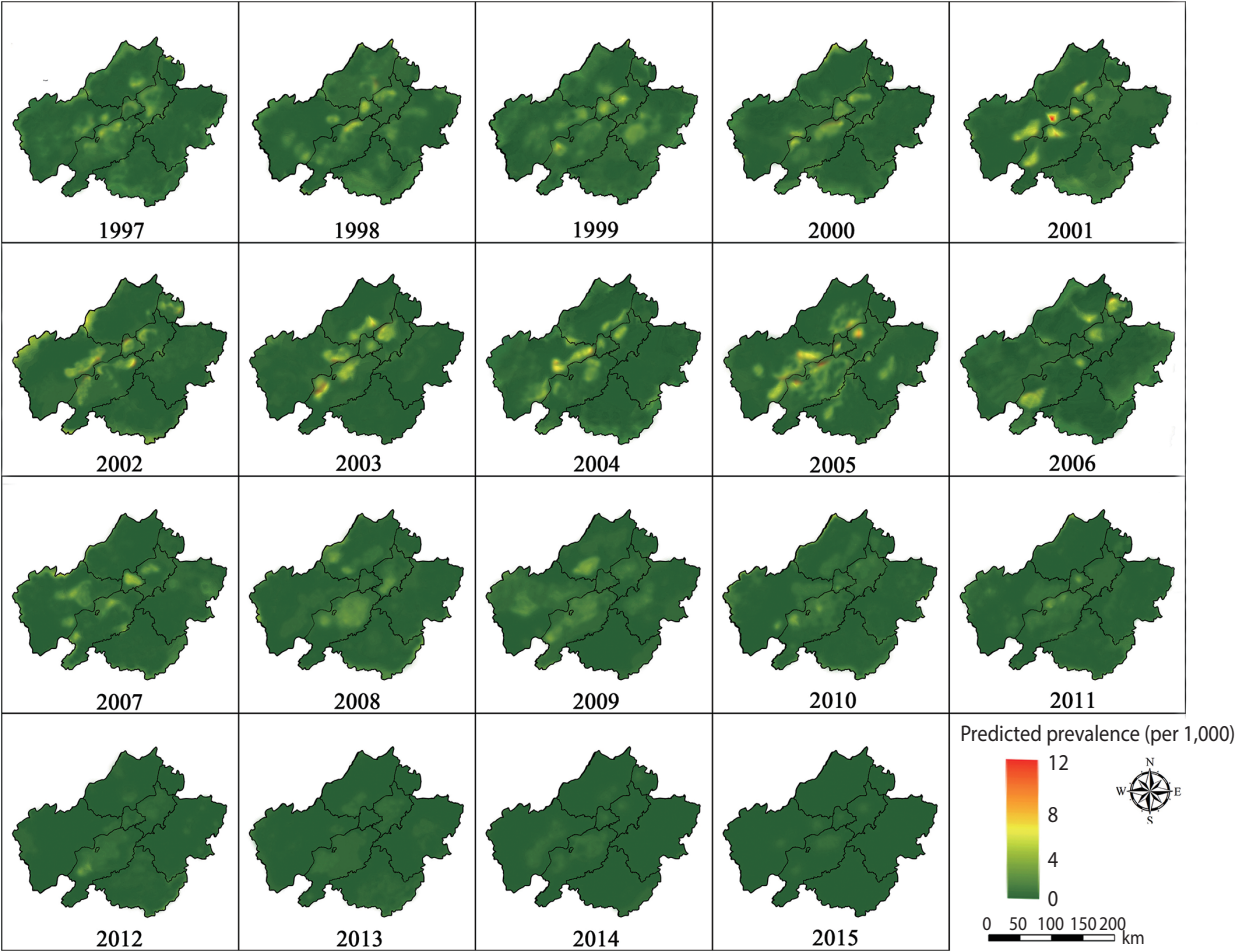
Plot for the annual prevalence of schistosomiasis predicted from 1997 to 2015 in Anhui Province, China.

**Figure 5.**
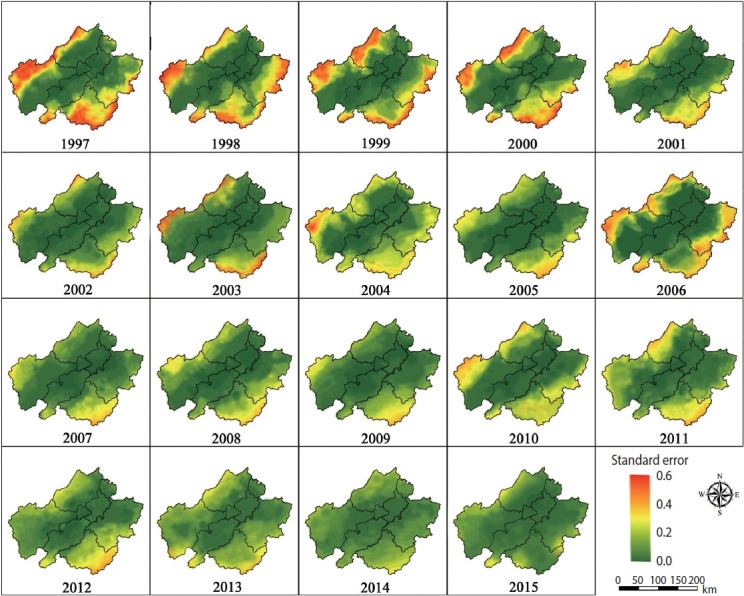
Plot for the annual standard error of the predicted prevalence of schistosomiasis from 1997 to 2015 in Anhui Province, China.

A map of the annual predicted prevalence for schistosomiasis is displayed in Figure 4. Starting in 1997, the prevalence was relatively high and showed a gradual increase, as indicated by the expanding yellow areas and the occasional red spots. The epidemic reached its peak in 2005, characterized by extensive light yellow and small red areas. Following this peak, the prevalence began to decline and remained relatively stable at a low level from 2006 to 2011. The predictions, represented by a dark green shade, showed a consistent uniformity across the study area, with values nearing zero from 2012 to 2015. Figure 5 illustrates the standard error of the corresponding estimates, indicating that the values were higher in areas where the sample villages were less densely distributed. However, the standard errors remained low throughout the study period.

## DISCUSSION

Our study presents a comprehensive application of advanced DL methods for quantifying local trends of schistosomiasis prevalence to assess the effectiveness of 2 NSCPs in the Yangtze River Basin, China. These estimates highlight substantial differences within the study area in levels and trends. The annual predicted prevalence map illustrates the disease’s progression, showing fluctuating trends until a relatively stable low level after 2011. These findings contribute to our understanding of schistosomiasis dynamics and control strategies currently implemented in China.

The environmental factors that affect snail habitats and the growth and reproduction of the snails have been confirmed in previous studies [12,24-26]. A study found that an ideal snail habitat is distributed less than 1 km from the water source [24]. In line with this, our study found a negative association between the proximity to water bodies and the prevalence of schistosomiasis. Increased rainfall can facilitate the dispersal of snails to new areas, including rivers, lakes, and wetlands [4]. The optimal survival temperature for the eggs of the parasite varies between 16°C and 35°C [27], a fact that supports the transmission of *S. japonicum* in snails that reproduce and grow under conditions of higher LST_day_ and therefore longer hours of daylight. Vegetation could reduce solar radiation and regulate the water temperature for host snails, thus providing comfortable spawning shelters [25]. As a result, a higher NDVI is more conducive to snail survival.

The results of model comparison indicated that our CNN-IDE was the optimal model that best accounted for the spatio-temporal variation in schistosomiasis prevalence. Figure 3 shows its superiority in predicting the schistosomiasis prevalence data, possibly because the complexity of the dynamics presented in a latent process can be captured flexibly if a sufficient number of parameters is available, and the CNN was trained on a massive amount of spatial data to obtain this [22]. Deep neural nets, especially CNNs, contain the necessary structure to harness this complexity. Furthermore, CNNs offer a global prior model for the dynamics that is both realistic and computationally efficient [22].

We found that the distribution patterns of schistosomiasis infection varied over space and time throughout the study period. These shifts in pattern can likely be attributed to changes in control strategies implemented at different stages of schistosomiasis management, potentially leading to a non-linear dynamic process in the prevalence of the disease [15,28]. The 10-year WBLP, launched in 1992, has effectively facilitated the praziquantel chemotherapy strategy [10]. Figure 4 shows that the predictions indicated schistosomiasis was maintained at a relatively stable and low level until 2001, after which there was a resurgence following the conclusion of the WBLP. This resurgence may be due to the fact that chemotherapy measures were limited to bovines and humans. Considering that over 40 species of mammals can act as potential zoonotic reservoirs, these measures are insufficient to completely interrupt the life cycle of the parasite [29]. Another possible explanation for the rebound is environmental and social factors, such as the floods of the Yangtze River in the late 1990s, ecological changes, and population movements [30,31].

To address this issue, a comprehensive national control strategy was put into place in 2005, encompassing agricultural mechanization, improved sanitation, and health education [32,33]. This strategy has been effective, leading to positive changes in both environmental conditions and human behaviors. Socioeconomic improvements, including better access to healthcare, enhanced sanitation facilities, and changes in water-related activities, have contributed to the reduction in disease transmission [34]. Furthermore, modifications in agricultural practices and water management may have impacted snail habitats [35]. The success of this comprehensive control strategy is evident based on the reduced number of schistosomiasis foci from 2006 to 2011, as shown in Figure 4. The sustained low prevalence of the disease from 2012 to 2015 further demonstrates the effectiveness and durability of these measures, underscoring the importance of continuing with this strategy.

Our CNN-IDE model has proven its superiority over traditional models in capturing the intricate spatio-temporal variations of schistosomiasis prevalence. This success is rooted in the model’s flexible ability to comprehend latent process complexities through an ample parameter set, bolstered by extensive training on spatial data using a CNN [24]. The CNN’s inherent ability to process complex multilayered information enables it to accurately represent the dynamics of schistosomiasis. This advantage highlights the potential of our model as a promising tool for future spatio-temporal risk modeling in schistosomiasis, contributing to the advancement of precision public health methodologies.

The limitations of the study need to be discussed. First, the low prevalence of schistosomiasis might result from the suboptimal specificity of serological analysis and sensitivity of stool examinations. It will be necessary to consider diagnostic errors in future modeling research to improve prediction accuracy. Second, we only considered a limited number of environmental factors and socioeconomic factors, such as household financial situations and medical conditions, were not included because these covariates were not available at the village level. Nevertheless, the random effect, *η_t_* (*s*), as shown in Supplementary Material 3: equqtion (2), is the “residual” after discounting what the covariates explain [23]. This suggests that covariates not included in our analysis are left in this “residual.” Third, we obtained data on water bodies in 2015, since historical and updated data on tributaries of the Yangtze River were not available.

In summary, the proposed CNN-IDE model effectively captured the complex dynamic process of schistosomiasis prevalence. The high-resolution 1× 1-km grid-level maps in our study facilitate the quantification of inequalities in prevalence to guide the efficient deployment of resources and interventions to those with the greatest need. As researchers, policymakers, and program implementors need to work together to achieve schistosomiasis elimination, our study provides a precision tool, guiding them where to go next.

## Figures and Tables

**Figure f6-epih-46-e2024039:**
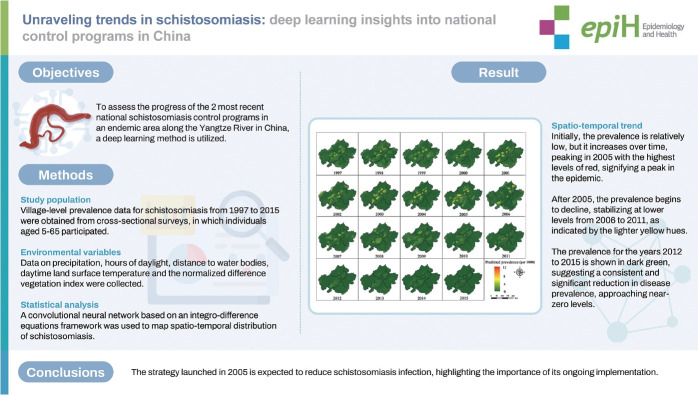


**Table 1.** Estimations of parameters for schistosomiasis in the CNN-IDE model

Parameters	Estimate	SE	z	p-value
Intercept	-2.76	1.23	-7.78	<0.01
Average daily precipitation	1.35E-05	1.63	2.26	0.02
Hours of daylight	-1.13E-03	0.42	-10.21	<0.01
Distance to water body	-8.44E-03	0.19	6.61	<0.01
LST_day_	-1.17E-05	1.12	-7.24	<0.01
NDVI	4.16E-06	0.10	-6.71	<0.01
θ_p,1_^1^	2.31E+02	10.11	1.89	0.21
θ_p,2_^2^	6.50E-03	13.01	2.01	0.14
θ_p,3_^2^	2.45E-02	10.92	3.92	0.04

CNN, convolutional neural network; IDE, integro-difference equation; SE, standard error; LST_day_, daytime land surface temperature; NDVI, normalized difference vegetation index.

1 Diffusion parameter.

2 Shift parameter.

## Data Availability

The datasets used and analyzed during the current study are available from the corresponding author on reasonable request and from a website repository (http://data.cma.cn/site/article/id/51.html, http://ladsweb.nascom.nasa.gov, http://worldwildlife.org).

## Supplementary materials

Supplementary Material 1. Information of villages and participants

Supplementary Material 2. Features of the environmental variables

Supplementary Material 3. The FRK model
