# Supplementary material for: Unraveling trends in schistosomiasis: deep learning insights into national control programs in China
Source: Epidemiol Health. 2024 Mar 13;46:e2024039. doi: 10.4178/epih.e2024039 (PMC11369565; doi:10.4178/epih.e2024039)
Supplement: Supplementary Material 1. — Information of villages and participants [file epih-46-e2024039-Supplementary-1.docx]

**Table S1 Information of villages and participants**

| **Year** | **Number of villages** | **Number of** **participants** | | | | |
| --- | --- | --- | --- | --- | --- | --- |
|  |  | **min** | **lower quartile** | **median** | **upper quartile** | **max** |
| 1997 | 1666 | 100 | 250 | 456 | 834 | 4021 |
| 1998 | 1670 | 100 | 270 | 496 | 834 | 4919 |
| 1999 | 1632 | 100 | 265 | 494 | 832 | 3683 |
| 2000 | **1683** | 100 | 258 | 513 | 845 | 3375 |
| 2001 | 1598 | 100 | 286 | 542 | 877 | 3535 |
| 2002 | 1498 | 100 | 240 | 502 | 907 | 3228 |
| 2003 | 1455 | 100 | 263 | 521 | 861 | 3204 |
| 2004 | 1522 | 100 | 340 | 654 | 1034 | 5852 |
| 2005 | 1208 | 100 | 375 | 829 | 1351 | 5401 |
| 2006 | 1164 | 100 | 500 | 910 | 1381 | 4948 |
| 2007 | 1178 | 100 | 521 | 905 | 1325 | 4334 |
| 2008 | 1200 | 100 | 514 | 930 | 1401 | 4285 |
| 2009 | 1067 | 100 | 525 | 905 | 1404 | 4786 |
| 2010 | 1200 | 100 | 617 | 971 | 1423 | 4374 |
| 2011 | 1234 | 100 | 626 | 963 | 1393 | 4709 |
| 2012 | 1296 | 100 | 595 | 911 | 1365 | 4820 |
| 2013 | 1067 | 100 | 595 | 933 | 1376 | 4700 |
| 2014 | 1142 | 100 | 607 | 933 | 1408 | 5801 |
| 2015 | **1028** | 100 | 582 | 951 | 1432 | 5946 |
