# Supplementary material for: Unraveling trends in schistosomiasis: deep learning insights into national control programs in China
Source: Epidemiol Health. 2024 Mar 13;46:e2024039. doi: 10.4178/epih.e2024039 (PMC11369565; doi:10.4178/epih.e2024039)
Supplement: Supplementary Material 2. — Features of the environmental variables [file epih-46-e2024039-Supplementary-2.docx]

**Table S2 Features of the environmental variables**

| **Data type** | **Source** | **Data period** | **Temporal resolution** | **Spatial resolution** |
| --- | --- | --- | --- | --- |
| Water bodies | SWBD^c^ | 2015 | - | 30m |
| LSTday^a^ | MODIS^d^ | 1997-2015 | Monthly | 1km |
| NDVI^b^ | MODIS^e^ | 1997-2015 | Monthly | 1km |
| Annual precipitation | Worldclim^f^ | 1997-2015 | Monthly | 1km |
| hours of daylight | Worldclim^f^ | 1997-2015 | Monthly | 1km |

^a^ Land surface temperature at day time.

^b^ Normalized difference vegetation index.

^c^ Shuttle Radar Topography Mission Water Body Data (SWBD).

^d^ Moderate Resolution Imaging Spectroradiometer (MODIS)/Terra, available at: http://modis.gsfc.nasa.gov/.

^e^ Moderate Resolution Imaging Spectroradiometer (MODIS)/Terra, available at: http://modis.gsfc.nasa.gov/.

^f^ Available at: http://www.worldclim.org/.
