# Supplementary material for: Unraveling trends in schistosomiasis: deep learning insights into national control programs in China
Source: Epidemiol Health. 2024 Mar 13;46:e2024039. doi: 10.4178/epih.e2024039 (PMC11369565; doi:10.4178/epih.e2024039)
Supplement: Supplementary Material 3. — The FRK model [file epih-46-e2024039-Supplementary-3.docx]

***The FRK model***

1. The data model: let ***Z*** *(s; t*) (a vector that contains both temporal and spatial dimensions) be the observed value of schistosomiasis japonica in village *s* at year *t*, which considered an additive measurement error. The data model used was expressed as:

$\boldsymbol{Z}(s; t)\boldsymbol{= Y}(s; t) + \varepsilon(s; t)$ (1)

where ***Y*** (*s; t*) is the latent process, and ε (*s; t*) is the observation errors independent of ***Y*** (*s; t*), which is assumed to be the independent identically distributed (iid) mean-zero measurement error; and

2. the process model was expressed by the equation:

$Y(s; t) = x(s; t)'\beta+\gamma t+ \eta(s; t)$ (2)

where *x* (*s; t*) is a fixed term indicating the covariant environmental factors, t is a temporal component, $\beta$ and $\gamma$ are the corresponding coefficients of each variable, and *η* (*s; t*) denotes a mean-zero random process that is dependent on space-time. Random effects can be divided into two parts, as follows:

$\eta(s; t) =\sum_{i=0}^{n} \phi_{i}(s;t)\alpha_{i}+v(s; t)$ (3)

where${\{\phi}_{i}\left( s;t \right), i=1,\ldots,\alpha_{i}$, corresponding to location (*s; t*), specifies a spatiotemporal basis function, {$\alpha_{i}$}, which are random effects explaining variations in schistosomiasis japonica risk, and $v\left( s; t \right)$ represents the small-scale spatiotemporal random effects not captured by the basis function. The generic basis function used by is the bisquare function given by:

$b(s, v)\left\{ \begin{aligned} {\{1-(||v-s||/r)}^{2}{\}}^{2}, ||v-s||\leq r, \\ 0 ,otherwise, \end{aligned} \right.$ (4)

where *r* is the aperture parameter, which is set automatically. $s$ and $v$ represent the positions of any two points.

***The IDE model***

To capture the spatiotemporal dynamic evolution of schistosomiasis japonica in the villages, we also constructed a dynamic model based on HM, as follows:

1. The data model:

$\boldsymbol{Z}_{t}=\boldsymbol{b}_{t}+\boldsymbol{H}_{t}\boldsymbol{Y}_{t}+\varepsilon_{t}, t=1,\ldots,T$ (5)

where $\boldsymbol{Z}_{t}$ is the data vector, including arbitrary position at time *t*; $b_{t}$ an additive offset term that describes the main trend of $\boldsymbol{Z}_{t}$ corresponding to non-dynamic spatio-temporal structure; $\boldsymbol{H}_{t}$ the observation mapping matrix which relates the process to the observations gained by mapping; $\boldsymbol{Y}_{t}$ the latent dynamic spatio-temporal process; and $\varepsilon_{t}$ an additive error process that is time varying (but statically dependent in time, assumed to be a mean-zero, Gaussian process); and

2. the process model, an approach represented by a first-order, integral-difference equation in a continuous spatial field. It is generally assumed that the process satisfies a Markovian spatio-temporal process, which indicates that its value at the current, given position is composed of two parts: a weighted combination of processes in the entire space domain at the previous time, and an additive, Gaussian and spatially coherent "innovation" expressed as:

$Y_{t}\left( s \right)=\int_{D_{s}} m(s,x;\theta_{p})Y_{t-1}\left( x \right)dx+\eta_{t}\left( s \right), s,x\in D_{s,} t=1,2\ldots,$ (6)

where $m\left( s,x;\theta_{p} \right)$is a transition kernel, which quantifies the evolution of process from the previous time to the current time, depending on the parameters $\theta_{p}$; and $\eta_{t}\left( s \right)$a mean-zero Gaussian spatial process independent of $Y_{t-1}\left( \cdot\right)$ those changes over time but is statistically independent. In a one-dimensional spatial domain, $m(s,x;\theta_{p})$ is assumed to be a Gaussian-shape kernel as a function of *x* relative to the location *s*:

$m\left( s,x;\theta_{p} \right)=\theta_{p,1}(s)exp(-\frac{1}{\theta_{p,2}\left( s \right)}[\left( x_{1}-\theta_{p,3}\left( s \right)-s_{1} \right)^{2}+\left( x_{2}-\theta_{p,4}\left( s \right)-s_{2} \right)^{2}]$(7)

***CNN-IDE model***

*Data level*

Assume the $\boldsymbol{Z}_{it}$ represents the observed prevalence of *S. japonicum* at an arbitrary spatial location $i$ and year *t*. The data model reflects the relationship between the observations and the underlying process:

$\boldsymbol{Z}_{it}=\boldsymbol{b}_{it}+\boldsymbol{H}_{it}\boldsymbol{Y}_{it}+\varepsilon_{it}, t=1,\ldots,T i=1, \ldots, I$ (8)

where $\boldsymbol{b}_{it}$ is an additive offset term including both intercept and fixed covariates (e.g., environmental factors); Term $\boldsymbol{H}_{it}$ is the incidence matrix which connects the observed data and the latent process $\boldsymbol{Y}_{it}$. The $\varepsilon_{t}$ is a time varying (but statistically temporally independent) continuous mean-zero Gaussian process.

*Process level*

The unobserved spatio-temporal process $\boldsymbol{Y}_{it}$ was modelled using a state-dependent model, which assumed that the spatially-varying dynamic process at time *t* was determined by the previous time points (denoted by set $\tau$ ) in a nonlinear fashion. The state-dependent process model can be described as:

$Y_{t}\left( s \right)=\int_{D_{s}} k(s,x;{\theta(s;y}_{t-1}^{(\tau)},\psi))Y_{t-1}\left( x \right)dx+\eta_{t-1}\left( s \right), s,x\in D_{s,} t=2,3\ldots,$ (9)

where $k(s,x;{\theta(s;y}_{t}^{(\tau)},\psi))$ is a state-dependent mixing kernel with unknown parameters $\psi$, and $\eta_{t}\left( s \right)$ a mean-zero Gaussian spatial error that is time independent. Crucially, we aimed to find the nonlinear relationship between the latent process $y_{t}^{(\tau)}\left( \cdot\right) \mathrm{and}$the spatially-varying parameters $\theta\left( \cdot\right)$ through some model parameterised by $\psi$. The mixing kernel can be determined by $y_{t}^{(\tau)}\left( \cdot\right)$ once the mapping is found and thus able to predict $Y_{t+1}\left( \cdot\right).$ We use a squared-exponential kernel [1], as shown below:

$k(s,x;{\theta(s;y}_{t}^{\left( \tau\right)},\psi))\equiv\frac{1}{{4{\pi\theta}_{1}(s;y}_{t}^{\left( \tau\right)},\psi)}\exp\left( -\frac{{h(s,x;{\theta(s;y}_{t}^{\left( \tau\right)},\psi))}^{2}}{{4\theta_{1}(s;y}_{t}^{\left( \tau\right)},\psi)} \right)$ (10)

where $\theta_{1}\left( \cdot\right)$ indicates the diffusivity (the spatially varying amplitude), and $\theta_{2}\left( \cdot\right)$ and $\theta_{3}\left( \cdot\right)$ describe the advection of process (shift).

*Encoding by CNN*

We used CNNs to encode the spatio-temporal dependency, which depends on the parameters vector $\theta\left( \cdot\right)$ that describe process diffusivity and advection. These features need to be identified and extracted from a series of process realization. The classic CNN is mainly composed of three parts: (1) a convolution layer, whose main function is to extract local features in the process; (2) a pooling layer, which can be used for dimensionality reduction; and (3) the full connection layer, which goes back to the principles of traditional neural networks, which is used to return the results as output [2]. The final output encoded the locations and strengths of the latent process dynamic.

The convolution layer: In signal processing, convolution is always used for signal detection. Consider a one-dimensional function *f* (·) to represent a signal and another one-dimensional function *g* (·) (also called a filter) to encode a feature. The convolution of *f* (·) and *g* (·) returns a function with a larger absolute value in the region where *f* (·) has similar characteristics to *g* (·), and a smaller absolute value in the other regions. The operation process of the convolution layer is shown in Supplementary Figure 1. This process can be understood as using a filter (convolution kernel) to categorize each small area of the image, so as to obtain their eigenvalues. In practical applications, there are often multiple convolution kernels. Supplementary Figure 2 applies 6 convolution kernels (filters) to the set of input images of size 64 × 64 each. After processing, images of size 32 × 32 are produced. This process can be repeated with more filters, where. the output of one becomes the input of the next.


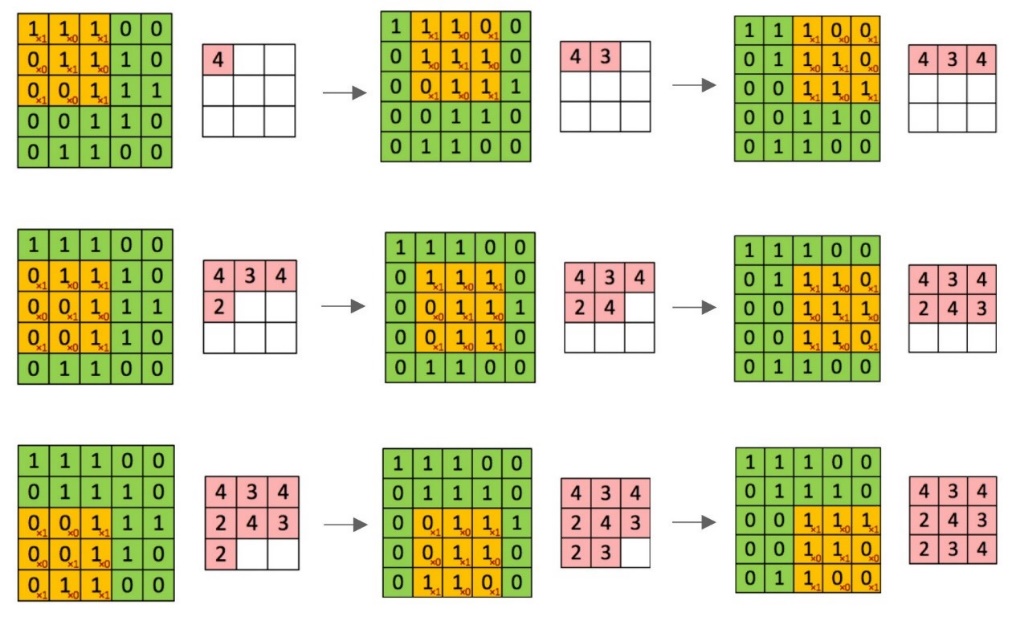


**Supplementary Figure 1** The operation process of the convolution layer.


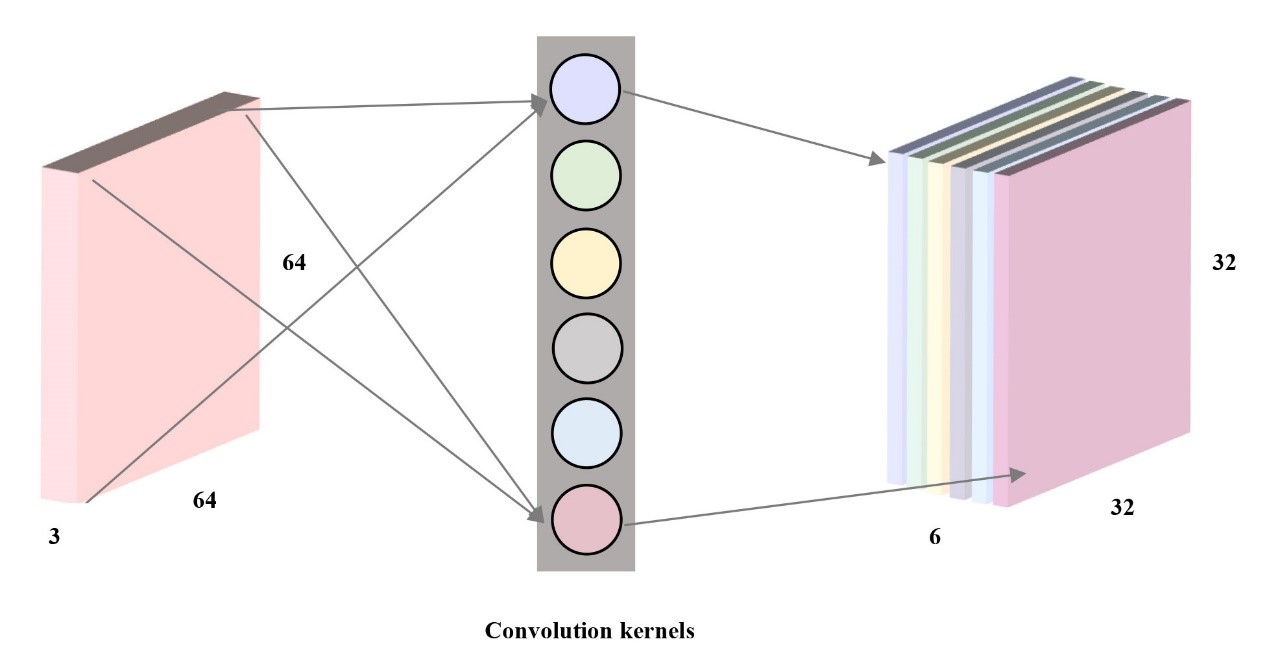


**Supplementary Figure 2** Convolution kernel of the convolution layer. 6 convolution kernels (filters) applied to the set of input images of size 64 × 64 each.

The pooling layer: pooling, also called down-sampling, is to extract the features obtained by convolution again to greatly reduce the data dimension, as shown in Supplementary Figure 3. Convolutions are followed by a rectified linear unit, and a pooling unit. The process includes maximum pooling and average pooling, where the former is used to extract the maximum and the latter to obtain the average. We generally use maximum pooling. The pooling layer can reduce data dimension more effectively than the convolution layer, which not only greatly reduces the amount of computation, but also effectively avoids over-fitting.


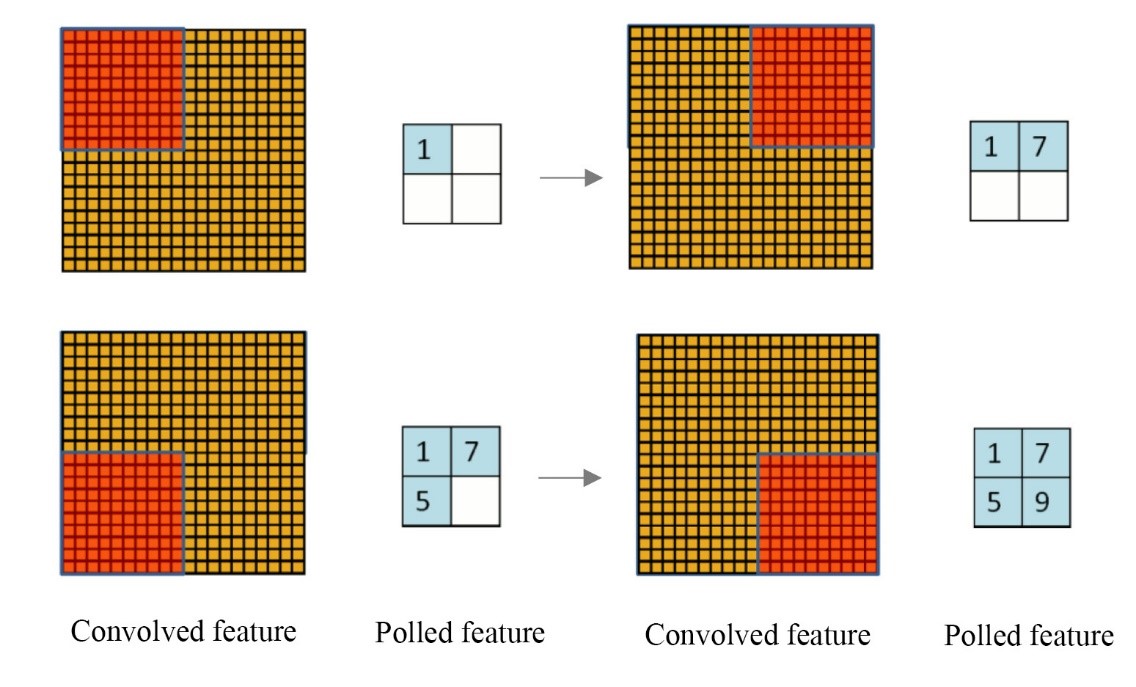


**Supplementary Figure 3** Pooling of the pooling layer. Also called down-sampling, is to extract the features obtained by convolution again to greatly reduce the data dimension.

The full connection layer: Only after reducing the dimension of the convolution layer and the pooling layer can the fully connected layer (similar to the traditional neural network (Supplementary Figure 4)) run, otherwise the large amount of data will lead to high computing cost and low efficiency. Supplementary Figure 5 shows the whole process of CNN. Convolution and pooling are repeated to reduce dimension as much as possible.


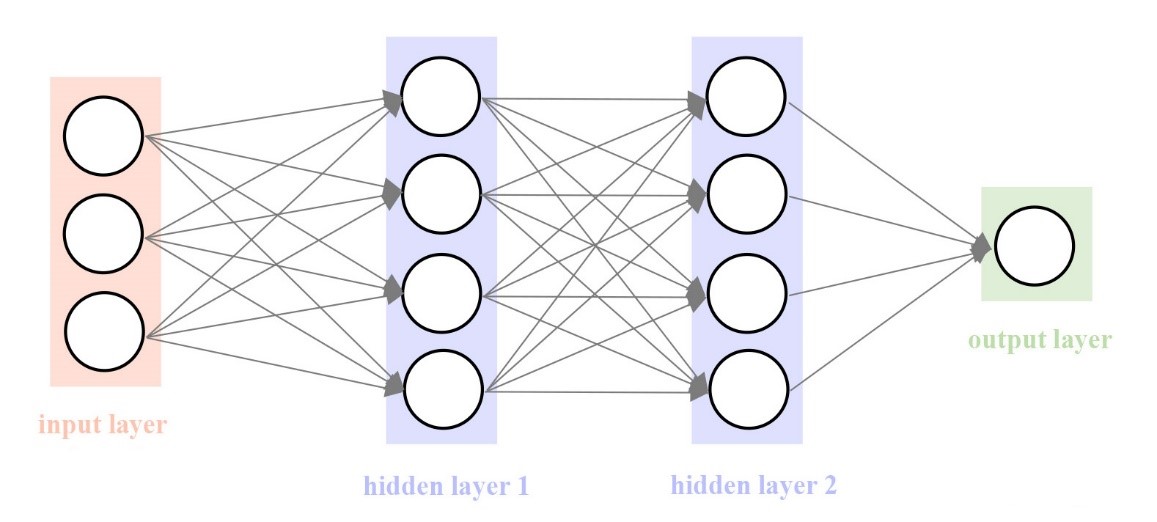


**Supplementary Figure 4** The fully connected layer. Similar to the traditional neural network, the input information is processed by several hidden layers and then output.


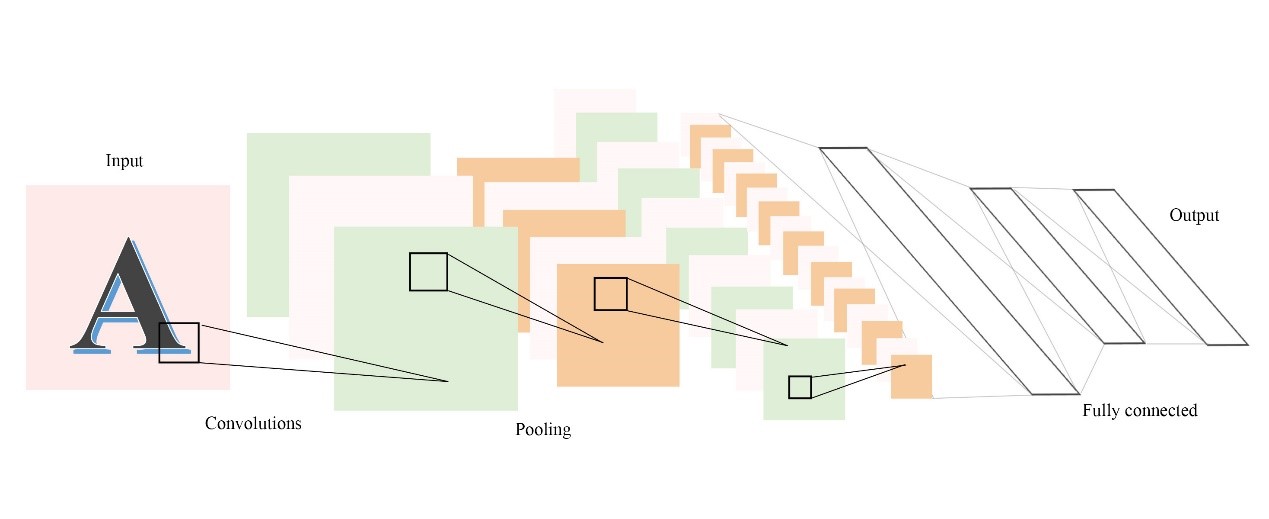


**Supplementary Figure 5** The whole process of CNN.

**Reference**

1. Wikle CK, Zammit-Mangion A, Cressie N. Spatio-temporal Statistics with R: CRC Press, Boca Raton, FL; 2019, p. 206-222.

2. Pourbabaee B, Roshtkhari MJ, Khorasani K. Deep convolutional neural networks and learning ECG features for screening paroxysmal atrial fibrillation patients. IEEE Transactions on Systems, Man, and Cybernetics: Systems 2018; 48:2095-2104.
